# A comparative study of percutaneous endoscopic interlaminar discectomy and transforaminal discectomy for L5-S1 calcified lumbar disc herniation

**DOI:** 10.1186/s12891-022-05186-z

**Published:** 2022-03-12

**Authors:** Yuan-Pei Cheng, Xiao-Kang Cheng, Han Wu

**Affiliations:** 1 Department of Orthopaedics, China-Japan Union Hospital of Jilin University, 130033, Jilin, China; 2 Department of Orthopaedics, Beijing Tongren Hospital Affiliated to Capital Medical University, 100730, Beijing, China

**Keywords:** Calcified lumbar disc herniation, Percutaneous endoscopic lumbar discectomy, Percutaneous endoscopic interlaminar discectomy, Percutaneous endoscopic transforaminal discectomy

## Abstract

**Background:**

Percutaneous endoscopic lumbar discectomy (PELD) is a relatively safe and effective minimally invasive surgery in the treatment of calcified lumbar disc herniation (CLDH). However, studies on percutaneous endoscopic interlaminar discectomy (PEID) and percutaneous endoscopic transforaminal discectomy (PETD) for CLDH have rarely been reported. This research aimed to compare the clinical efficacy of PEID and PETD for L5-S1 CLDH.

**Methods:**

We retrospectively analyzed 54 consecutive patients with L5-S1 CLDH treated with PELD at our institution from August 2016 to August 2020. Patients were divided into PEID group (*n* = 28) and PETD (*n* = 26) group according to the surgical methods. The demographic characteristics and surgical results of the two groups were compared. Clinical outcomes were estimated by the visual analog scale (VAS) for leg pain, Oswestry disability index (ODI) and modified MacNab criteria.

**Results:**

All patients were successfully operated on by PEID or PETD. No significant differences in the demographic characteristics, intraoperative blood loss, postoperative hospital stay and complication rate were noted between the PEID and PETD groups. The excellent and good rates in the PEID group were similar to those in the PETD group (89.29% vs 88.46%, *P *= 1.000), whereas the PEID group exhibited superior results for operative time (min) (64.61 ± 5.60 vs 85.58 ± 8.52, *P* < 0.001) and fluoroscopy times (n) (2.93 ± 0.90 vs 13.35 ± 2.30, *P* < 0.001) compared with the PETD group.

**Conclusions:**

PEID has achieved good clinical efficacy as PETD for L5-S1 CLDH. Compared with PETD, PEID has the advantages of shorter operative time and a reduced number of fluoroscopy times in the treatment of CLDH.

## Background

Calcified lumbar disc herniation (CLDH), with a low incidence rate, is a special type of lumbar disc herniation (LDH). Calcified disc herniation, with hard structure, usually adheres extensively to surrounding tissues such as nerve roots and the dural sac. Most patients with CLDH have severe low back and leg pain symptoms and even severe neurological symptoms in the acute stage [1]. However, conservative treatments fail to effectively relieve symptoms.

Patients with CLDH are typically treated by traditional open surgery. Traditional open surgery completely resects the calcified intervertebral disc with good clinical outcomes. However, traditional open surgery, with a long incision, extensive stripping of the paravertebral muscles and laminectomy, has some deficiencies, such as significant tissue damage, considerable intraoperative blood loss, muscle denervation and atrophy, and even spinal instability [2, 3].

Percutaneous endoscopic lumbar discectomy (PELD), including percutaneous endoscopic interlaminar discectomy (PEID) and percutaneous endoscopic transforaminal discectomy (PETD), is a minimally invasive operation for the treatment of LDH. Some studies demonstrated that PELD had similar clinical results as traditional open discectomy [4, 5]. PELD has less intraoperative blood loss, less trauma, faster postoperative recovery, and shorter hospital stay than traditional open surgery. However, it is difficult and challenging to treat CLDH with PELD because the calcified disc tightly adheres to nerve roots and the dural sac [6]. In recent years, with the appearance of surgical instruments such as ultrasonic osteotomes and endoscopic grinding drills, PELD has been gradually applied to the treatment of CLDH. However, there are no studies and reports comparing the efficacy of PEID and PETD in the treatment of L5-S1 CLDH. The research aims to compare the clinical effect of the two surgical approaches and to provide clinical guidance for L5-S1 CLDH.

## Methods

### General Information

This research obtained the support of the Ethics Committee of our institution and informed consent of all patients. From August 2016 to August 2020, we collected 54 consecutive patients with L5-S1 CLDH treated with PEID and PETD in our hospital. These patients were divided into PEID (*n* = 28) and PETD (*n* = 26) groups according to the surgical methods. The inclusion criteria were as follows: 1) symptoms of pain and numbness of lower limbs; 2) L5-S1 CLDH confirmed by preoperative lumbar X-ray, computed tomography (CT) and magnetic resonance imaging (MRI); 3) failure of conservative treatment or no significant improvement in symptoms for greater than 3 months; and 4) the follow-up time was greater than 12 months. The exclusion criteria were as follows: 1) multisegmental lesions; 2) noncalcified lumbar disc herniation; 3) lumbar spinal stenosis, lumbar instability, lumbar tuberculosis, lumbar infection or spinal tumor. A percutaneous transforaminal endoscopic spine system (Joimax, Karlsruhe, Germany), tip-flexible bipolar radiofrequency system (Elliquence LLC, USA), ultrasonic osteotome (SMTP, China) and endoscopic instruments (MaxMorespine GmbH, Germany) were used.

## Surgical Procedure

### PEID

**Fig.1:**
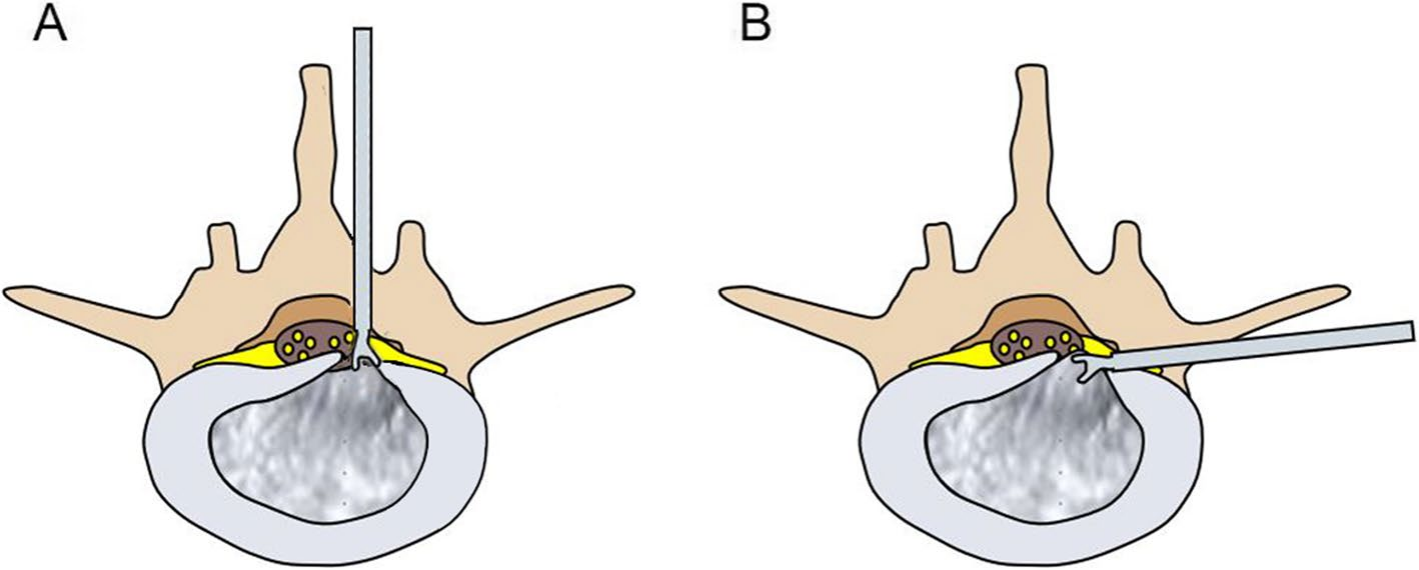
Schematic diagrams of PEID and PETD (A-B). **A** The schematic diagram of PEID. **B** The schematic diagram of PETD

**Fig.2:**
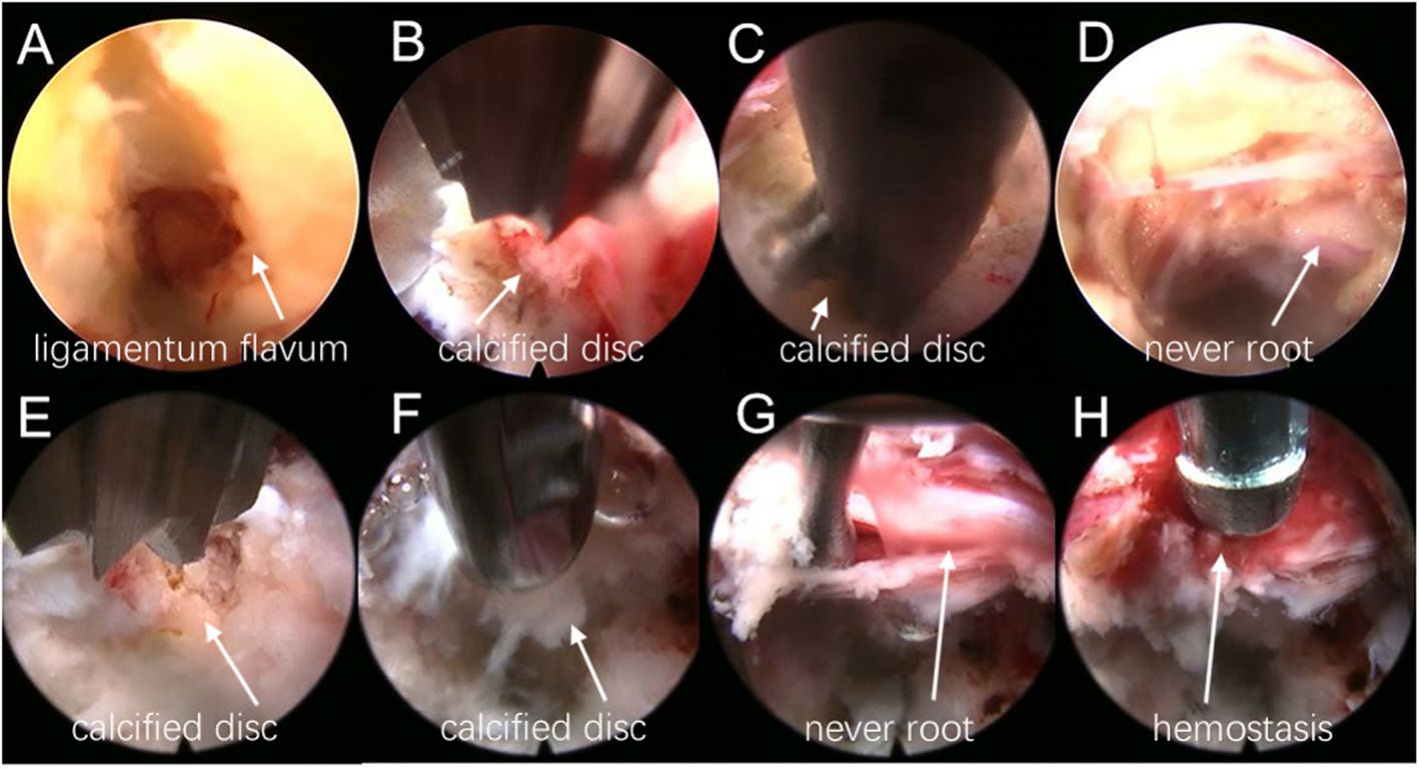
Endoscopic images of PEID (A-D) and PETD (E–H). **A** The ligamentum flavum was cut by scissors. **B** The calcified and herniated disc tissue was cut by an ultrasonic osteotome. **C** The calcified and herniated disc were taken out by a nucleus pulposus forceps. **D** The nerve root and dural sac were fully decompression. **E** An ultrasonic osteotome was used to cut the calcified disc. **F** A nucleus pulposus forceps was used to remove the calcified and herniated disc. **G** The nerve roots and dural sac were moved. **H** A bipolar radiofrequency was used to hemostasis

**Fig.3:**
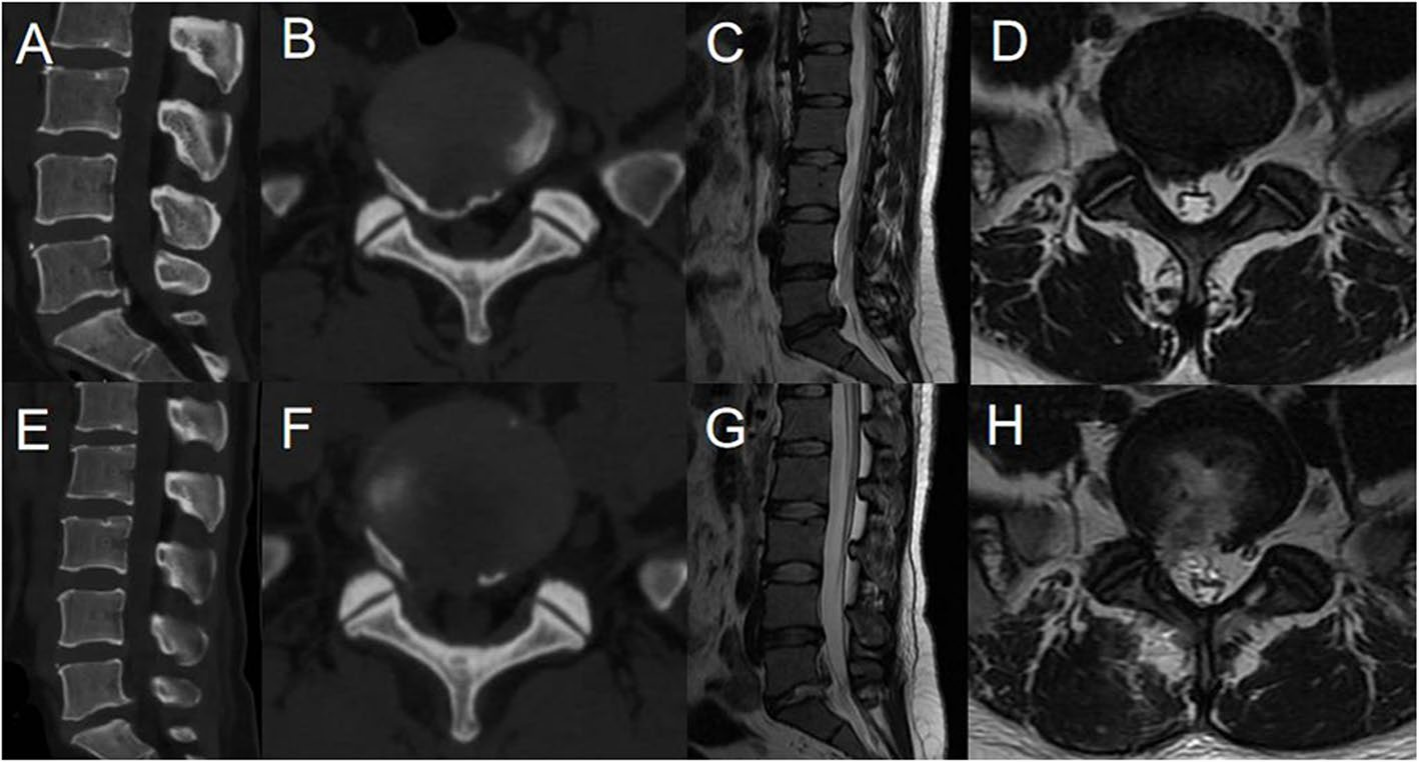
Pre- and post-operative MRI and CT (A-H). **A-D** Preoperative CT and MRI revealed lumbar disc herniation combined with calcification. **E–H** Postoperative CT and MRI showed that the calcified intervertebral disc was removed and the compressed nerve root had been relieved by PEID

All patients were placed on the operating table in the prone position after general anesthesia. The surgical segment was identified by fluoroscopy on an X-ray machine. After marking the puncture site on the body surface, routine disinfection was performed. The puncture point was anesthetized with local infiltration of 0.5% lidocaine. The puncture needle was inserted in 1.0 cm beside the posterior midline. The needle position was confirmed by fluoroscopy of a C-arm X-ray machine. A 0.7-cm longitudinal incision was made at the skin mark. The skin, subcutaneous tissue and lumbodorsal fascia were incised in turn. The dilating tube was placed to the ligamentum flavum. The working cannula and endoscope were inserted along the dilating tube (Fig. 1A). After checking the operating system, the light source was connected, and the operation was performed under continuous saline irrigation. Under the endoscope, the ligamentum flavum was cut by scissors (Fig. 2A). After reaching the epidural space, the lateral dural sac and nerve root were exposed. A blunt dissector was used to expose and separate the calcified disc and herniated nucleus pulposus. The locations of the nerve root, dural sac and herniated disc were explored. The degree of calcification and size of the herniated disc were carefully confirmed. Under the endoscope, the calcified and herniated disc tissue was removed by an ultrasonic osteotome (SMTP, China) (Fig. 2B), and the herniated free nucleus pulposus was removed using nucleus pulposus forceps (Fig. 2C). Under direct vision, no obvious compression of the nerve root and dural sac was observed (Fig. 2D). During the operation, bipolar radiofrequency ablation was used to adequately stop bleeding. After removing the working cannula, the incision was closed intradermally and covered with a sterile dressing. All patients underwent CT and MRI examinations before (Fig. 3A-D) and after (Fig. 3E-H) operation.

### PETD

**Fig.4:**
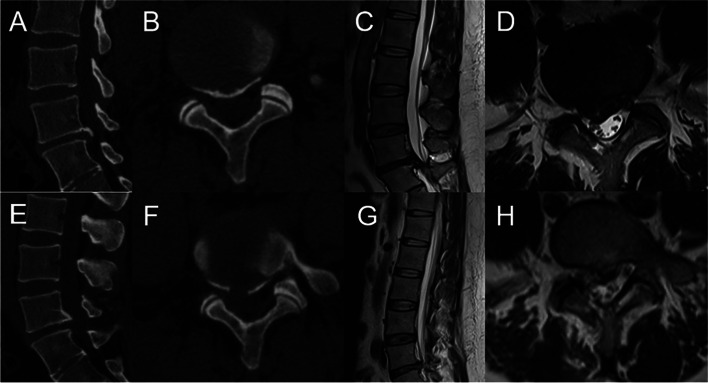
Pre- and post-operative MRI and CT (A-H). **A-D** Preoperative CT and MRI showed lumbar disc herniation combined with calcification. **E–H** Postoperative CT and MRI revealed the loosened nerve root after the calcified disc was removed by PETD

The patient was placed in a prone position after local anesthesia, and the responsible segment was identified by C-arm X-ray fluoroscopy. The level of responsible intervertebral space and the posterior midline line were marked. Routine disinfection and sheet laying were performed. The puncture site was set at 12 ~ 14 cm next to the posterior midline, and the puncture point was anesthetized with 0.5% lidocaine injection. The 18-G puncture needle was inserted in the marked puncture site, and the needle slid along the lateral aspect of the superior articular eminence and entered the spinal canal through the intervertebral foramen. It was confirmed that the puncture needle was located in the midline on the anterior radiograph and the posterior upper edge of the vertebral body on the lateral radiograph by fluoroscopy with a C-arm X-ray machine. After inserting the guide wire, a puncture needle was removed. The skin was cut to approximately 0.7 cm, and the expanding cannula was inserted gradually. Finally, the bevelled cannula was inserted along the dilatation tube. When the working channel was difficult to insert, foraminoplasty was performed, in which by a trephine was used to remove the anterior part of the superior articular process. Then the working cannula was successively introduced. The position of the working cannula was confirmed under fluoroscopy on a C-arm X-ray machine (Fig. 1B). The working cannula was flushed with 3000 ml of saline. A blunt dissector was used to reveal and separate the calcified disc and herniated nucleus pulposus. The calcified disc was cut with an ultrasonic osteotome (SMTP, China) (Fig. 2E). Subsequently, a nucleus pulposus forceps was used to remove the calcified disc and herniated nucleus pulposus under an endoscope (Fig. 2F). The working cannula was moved appropriately. The nerve roots and dural sac were moved by a blunt dissector (Fig. 2G). The nerve roots and dural sac were then explored for relaxation, and no protruding discs compressed the nerve roots and dural sac. The bipolar radiofrequency ablator was used to perform fibrous annuloplasty. Bleeding points were carefully explored and given adequate hemostasis (Fig. 2H). The nerve root and dural sac were confirmed to be free of compression, and no bleeding points were evident. The working cannula was removed. Then, the incision was sutured intradermally and covered with a sterile dressing. The CT and MRI examinations of all patients were done before (Fig. 4A-D) and after (Fig. 4E-H) operation.

## Measures

Demographic characteristics, such as age, sex, body mass index (BMI), duration of symptoms, type of calcification, location of calcification and follow-up time, were compared between the PEID and PETD groups. Surgical outcomes, such as operative time, intraoperative blood loss, intraoperative fluoroscopy times, postoperative hospital stay and complications, were compared between the two groups. Clinical outcomes indicators, including the preoperative and postoperative visual analog scale (VAS) [7] for leg pain, Oswestry disability index (ODI) [8], and modified MacNab criteria [9] at the last follow-up, were compared between the two groups.

## Statistical Assessments

The SPSS 25.0 program (IBM Corporation, USA) was used for statistical analysis of all data. Comparisons between the two groups were analyzed by independent-sample t tests or Wilcoxon test, whereas paired-samples t tests were used for intragroup comparison. Sex, type of calcification, location of calcification and complications were compared by chi-square tests. The modified MacNab criteria were compared by the Mann–Whitney U test. P < 0.05 indicates a statistically significant difference.

## Results

### Demographic characteristics and surgical outcomes

All patients were successfully operated on by a trained and experienced spine surgeon in our hospital. We followed up all 54 patients (28 in the PEID group and 26 in the PETD group). The demographic characteristics (age, sex, BMI, duration of symptoms, type of calcification, location of calcification and follow-up time) of the two groups were significantly similar as shown in Table 1. In the PEID group, the operative time, intraoperative fluoroscopy times, postoperative hospital stay, intraoperative blood loss and complication rate were 64.61 ± 5.60 min, 2.93 ± 0.90 times, 2.57 ± 1.14 days, 13.75 ± 4.44 ml and 7.14%, respectively. In the PETD group, the operative time, intraoperative fluoroscopy times, postoperative hospital stay, intraoperative blood loss and complication rate were 85.58 ± 8.52 min, 13.35 ± 2.30 times, 2.58 ± 1.07 days, 13.65 ± 3.62 ml and 7.69%, respectively. PEID was superior to PETD in terms of operative time and intraoperative fluoroscopy times, but no significant difference in intraoperative blood loss, postoperative hospital stay or incidence of complications were noted as shown in Table 2.

**Table 1:** Demographic characteristics of both the PEID group and PETD group

Variables	PEID (*n* = 28)	PETD (*n* = 26)	*P* Value
Age (years)	37.46 ± 6.68	37.73 ± 8.33	0.897
Sex			0.761
Male	15	15	
Female	13	11	
BMI (Kg/m^2^)	26.12 ± 3.27	26.01 ± 3.18	0.893
Duration of symptoms (months)	15.57 ± 18.61	15.46 ± 19.21	0.787
Type of calcification			0.310
Isolated type (< 3 mm)	11	6	
Half-lunar type (3–10 mm)	10	9	
Successive type (> 10 mm)	7	11	
Location of calcification			0.884
Central type	12	10	
Paracentral type	10	11	
Foraminal type	6	5	
Follow-up (months)	15.93 ± 3.03	15.73 ± 3.71	0.547

Values are mean ± SD, number, or as otherwise indicated. PEID, percutaneous endoscopic interlaminar discectomy; PETD, percutaneous endoscopic transforaminal discectomy; BMI, body mass index

**Table 2:** Surgical outcomes of both the PEID group and PETD group

Variables	PEID (*n* = 28)	PETD (*n* = 26)	*P* Value
Operative time (minutes)	64.61 ± 5.60	85.58 ± 8.52	< 0.001*
Fluoroscopy times (n)	2.93 ± 0.90	13.35 ± 2.30	< 0.001*
Intraoperative blood loss (ml)	13.75 ± 4.44	13.65 ± 3.62	0.866
Postoperative hospital stay (days)	2.57 ± 1.14	2.58 ± 1.07	0.985
Complications	2/26	2/24	1.000
Modified Macnab criteria			1.000
Excellent	14	13	
Good	11	10	
Fair	3	2	
Poor	0	1	

Values are mean ± SD, number, or as otherwise indicated. PEID, percutaneous endoscopic interlaminar discectomy; PETD, percutaneous endoscopic transforaminal discectomy

^*^ P < 0.05 versus PETD group

## Clinical outcomes

VAS for leg pain and ODI scores were used to evaluate the clinical results before and 1 day, 1 month, 3 months, 6 months and 12 months after the operation. The postoperative VAS and ODI scores of the two groups were obviously lower than those before surgery as shown in Table 3. No significant difference was found in VAS and ODI scores between the two groups at any follow-up time point before and after surgery as shown in Table 3. On the basis of the modified MacNab criteria at the last follow-up, no significant difference was found in the excellent and good rates of the two groups (89.29% vs 88.46%, *P* = 1.000) as shown in Table 2.

**Table 3:** VAS and ODI scores of both the PEID group and PETD group

Variables	PEID (*n* = 28)	PETD (*n* = 26)	*P* Value
VAS			
Preoperative	7.64 ± 0.91	7.54 ± 0.91	0.675
1 Day	2.96 ± 0.58*	3.19 ± 0.63*	0.172
1 Month	2.46 ± 0.51*	2.73 ± 0.67*	0.103
3 Months	2.18 ± 0.48*	2.38 ± 0.64*	0.187
6 Months	1.75 ± 0.44*	1.88 ± 0.52*	0.306
12 Months	1.46 ± 0.51*	1.65 ± 0.49*	0.167
ODI (%)			
Preoperative	70.79 ± 9.15	71.31 ± 10.20	0.844
3 Months	23.64 ± 3.93*	25.54 ± 4.74*	0.115
6 Months	19.43 ± 3.77*	21.31 ± 4.77*	0.113
12 Months	16.50 ± 3.76*	18.00 ± 3.96*	0.159

Values are mean ± SD, number, or as otherwise indicated

PEID, percutaneous endoscopic interlaminar discectomy; PETD, percutaneous endoscopic transforaminal discectomy; VAS, visual analog scale; ODI, Oswestry Disability Index

^*^ P < 0.05 versus preoperative

## Complications

In the PEID group, 1 patient had a dural sac tear but no cerebrospinal fluid leakage; 1 patient had postoperative dysesthesia. In the PETD group, 1 patient had postoperative dysesthesia; 1 patient had residue of herniation. The symptoms of patient with residue were relieved after the second operation while the rest of patients were cured after conservative treatments. During the follow-up period, there were no reports of epidural hematoma, infection or lower extremity deep vein thrombosis.

## Discussion

Although both PEID and PETD could achieve good clinical efficacy in the treatment of L5-S1 CLDH, our study showed that PEID group had shorter operative time and fewer intraoperative fluoroscopy times compared with the PETD group.

Compared with children, CLDH is more common in adults. Children usually are treated by conservative treatments, while adults fail to. Calcified disc herniation adheres extensively to nerve roots and the dural sac, which not only increases the difficulty of removal by PELD, but also enlarges the risk of nerve root injury and dural sac tear. Some previous studies showed that PELD was applied to treat CLDH [10–14]. Dabo et al. [15] showed that 30 patients of CLDH were treated by PEID with a trephine instrument, but 16 patients had postoperative dysesthesia 3 months after operation. Yu et al. [16] reported that the symptoms of 25 CLDH patients treated by PETD were relieved, but 7 patients had postoperative dysesthesia and 1 patient had recurrence of herniation.

In our study, postoperative outcomes demonstrated that the symptoms of all patients were significantly relieved by PEID or PETD. For the treatment of CLDH, we used an ultrasonic osteotome to remove the calcified disc. Ultrasonic osteotome had selective osteotomy properties and retains the adjacent soft tissue [17, 18]. Compared with grinding drills, ultrasonic osteotome was considered to be safe, accurate and effective for the removal of bone tissue [19]. Calcified intervertebral disc, nerve roots and dural sac should be carefully separated and exposed during the procedure. Care should be taken when pulling the nerve root and dural sac. Part of the soft herniated intervertebral disc should be taken out to create a large enough safe space. Small pieces of calcified intervertebral disc could be removed directly. Large calcified disc could be first divided into small pieces with an ultrasonic osteotome and removed in turn. The above measures can reduce the risk of the injury of nerve root and dural sac. Both PEID and PETD may be safer and more effective surgical methods for olderly, obviously frail or economically difficult CLDH patients.

Compared with PEID, PETD has some deficiencies in the treatment of L5-S1 CLDH. PETD is a relatively complex procedure with a long learning curve. Multiple punctures and adequate foraminoplasty are needed, especially in the L5-S1 level with foraminal stenosis and a high iliac crest, which not only increases the operative time and fluoroscopy times, but also enlarges the risk of the exiting nerve root injury. Moreover, surgeons and patients are also exposed to much radiation [20]. The working channel is not flexible enough during the procedure of PETD, which makes it difficult to sufficient decompression of the herniated calcified disc. Nie et al. [21] found that the operative time and fluoroscopy times of PEID were significantly shorter than those of PETD. The results were similar to those of the present study.

In our research, no significant difference was observed in the complication rate between the PEID and PETD groups, which may be due to the small number of cases or short follow-up time. In the PEID group, 1 patient had a dural sac tear but no cerebrospinal fluid leak, and 1 patient had postoperative dysesthesia. In addition, 1 patient had postoperative dysesthesia, and 1 patient had residue of herniation in the PETD group. According to our experience, repeated punctures and foraminoplasty may cause the nerve root injury, which may lead to postoperative dysesthesia. The tight adhesion between calcified intervertebral disc and surrounding nerve tissues may result in nerve root injury, dural sac tear and even cerebrospinal fluid leakage. Residue of herniation may be caused by the poor position of the working channel due to the obstruction of the high iliac crest, resulting in insufficient decompression.

There were some limitations in our research. First, this was a retrospective study. Moreover, the sample size is small and the follow-up period is short. Further studies with multicenter, large sample and long-term follow-up will be conducted in the future.

## Conclusions

Both PEID and PETD has achieved good clinical outcomes for L5-S1 CLDH. The clinical efficacy of PEID and PETD in the treatment of CLDH at L5-S1 level are comparable. Compared with PETD, PEID has the advantages of shorter operative time and a reduced number of fluoroscopy times in the treatment of L5-S1CLDH.

## Data Availability

The corresponding author could provide the date of the study if necessary.

## Abbreviations

CLDH: Calcified lumbar disc herniation
LDH: Lumbar disc herniation
MRI: Magnetic resonance imaging
CT: Computed tomography
PELD: Percutaneous endoscopic lumbar discectomy
PEID: Percutaneous endoscopic interlaminar discectomy
PETD: Percutaneous endoscopic transforaminal discectomy
